# A computed tomography‐based survey of paramedullary diverticula in extant Aves

**DOI:** 10.1002/ar.24923

**Published:** 2022-04-07

**Authors:** Jessie Atterholt, Mathew J. Wedel

**Affiliations:** ^1^ Graduate College of Biomedical Sciences Western University of Health Sciences Pomona California USA; ^2^ College of Osteopathic Medicine of the Pacific & College of Podiatric Medicine Western University of Health Sciences Pomona California USA

**Keywords:** air sacs, avian respiratory system, diverticula, osteological correlates, pneumaticity

## Abstract

Avian respiratory systems are comprised of rigid lungs connected to a hierarchically organized network of large, regional air sacs, and small diverticula that branch from them. Paramedullary diverticula are those that rest in contact with the spinal cord, and frequently invade the vertebral canal. Here, we review the historical study of these structures and provide the most diverse survey to date of paramedullary diverticula in Aves, consisting of observations from 29 taxa and 17 major clades. These extensions of the respiratory system are present in nearly all birds included in the study, with the exception of falconiforms, gaviiforms, podicipediforms, and piciforms. When present, they share connections most commonly with the intertransverse and supravertebral diverticula, but also sometimes with diverticula arising directly from the lungs and other small, more posterior diverticula. Additionally, we observed much greater morphological diversity of paramedullary airways than previously known. These diverticula may be present as one to four separate tubes (dorsal, lateral, or ventral to the spinal cord), or as a single large structure that partially or wholly encircles the spinal cord. Across taxa, paramedullary diverticula are largest and most frequently present in the cervical region, becoming smaller and increasingly absent moving posteriorly. Finally, we observe two osteological correlates of paramedullary diverticula (pneumatic foramina and pocked texturing inside the vertebral canal) that can be used to infer the presence of these structures in extinct taxa with similar respiratory systems.

## 1 INTRODUCTION

**FIGURE 1:**
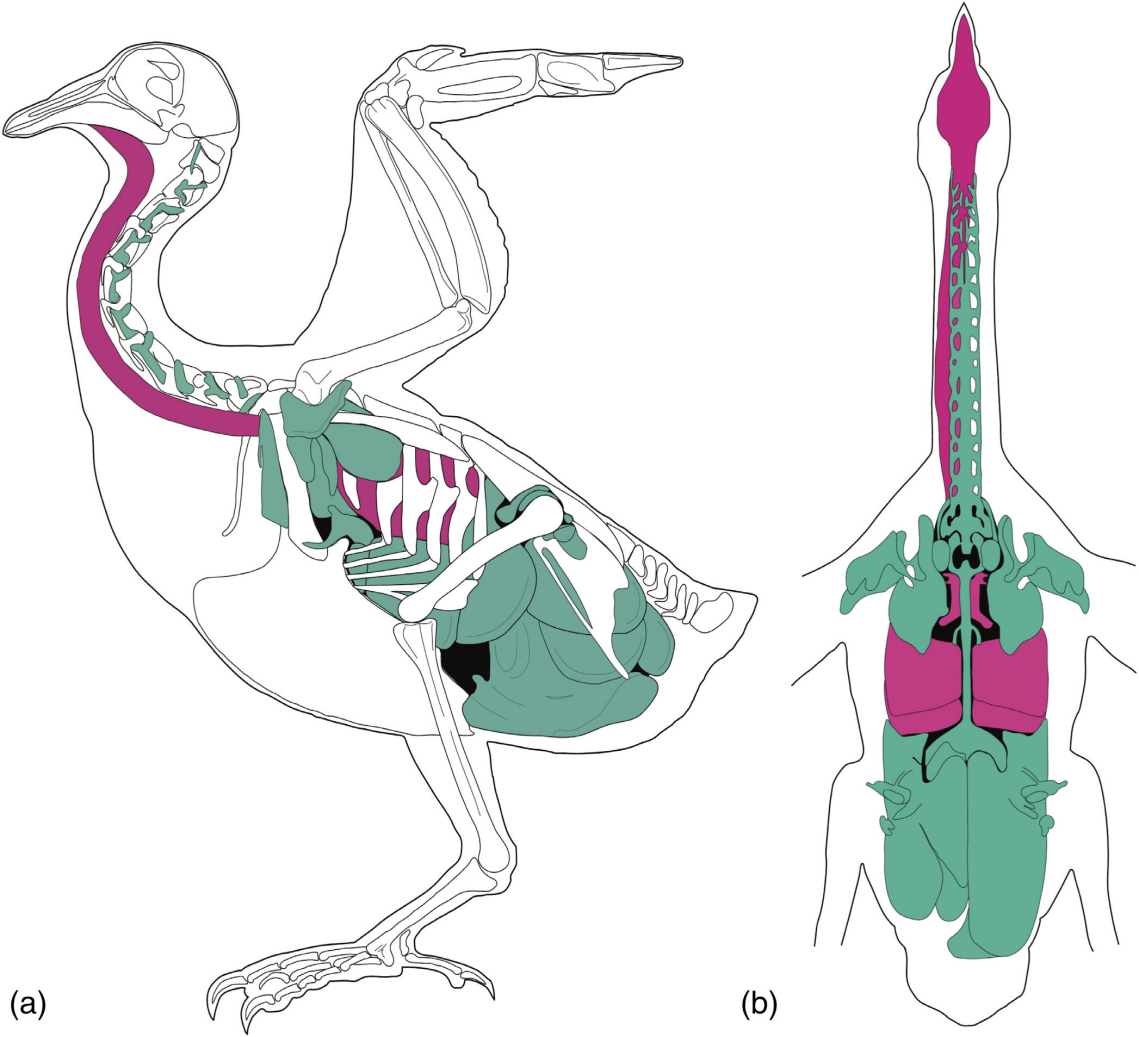
Diagrams of the avian respiratory system. Drawings show the respiratory system in a pigeon, in lateral (a) and dorsal (b) views; pink = trachea and lungs; green = air sacs and diverticula (Redrawn from Müller, 1908)

The respiratory system of modern birds, consisting of a pair of small, rigid lungs connected to an elaborate system of air sacs that pervade the body, has been described in detail in a number of living taxa (Baer, 1896; Bezuidenhout et al., 1999; Campana, 1875; Duncker, 1971; Hogg, 1984; King & Kelly, 1956; Müller, 1908; O'Connor, 2006) (Figure 1). This system of air sacs is organized hierarchically, with large, regional sacs branching into smaller diverticula, which in turn divide into smaller, often‐anastomosing, units. The air sac system permeates the entire body to a greater or lesser degree, invading bones, spaces between muscles, and spaces under the skin.

The vertebrae of birds are pneumatized by diverticula of the cervical and thoracoabdominal air sacs, as well as diverticula that emanate directly from the lung. In general, diverticula of the cervical air sacs pneumatize the cervical and anterior dorsal vertebrae, diverticula of the lungs pneumatize the mid‐dorsals, and diverticula of the abdominal air sacs pneumatize the posterior dorsal vertebrae, synsacrum, and caudal vertebrae (Bezuidenhout et al., 1999; Cover, 1953; Hogg, 1984; King, 1966; Müller, 1908; O'Connor, 2006; O'Connor & Claessens, 2005). These diverticula follow nerves and blood vessels as they spread along the vertebral column. The main cervical diverticula, the *canali intertransversarii* (intertransverse canals), follow the vertebral arteries and pass through the transverse foramina of the cervical vertebrae (Müller, 1908). Other diverticula have been observed on the anterodorsal surface of the vertebrae, forming supravertebral diverticula (Cover, 1953). In some instances, these connect with the intertransverse diverticula, via anterior diverticular prolongations (Cover, 1953).

Branches of the intertransverse diverticula may extend medially into the intervertebral foramina, where they contact the spinal cord and enter the neural canal, forming structures that have been called supramedullary diverticula (Müller, 1908; O'Connor, 2006). Occasionally, these diverticula also merge with the supravertebral diverticula. This elaborate and highly variable network of anastomosing diverticula around the outside of the vertebra and inside of the vertebral canal may even extend inside the bone of the vertebral body and arch. However, supramedullary diverticula are poorly understood, and have not been the subject of much previous study.

**FIGURE 2:**
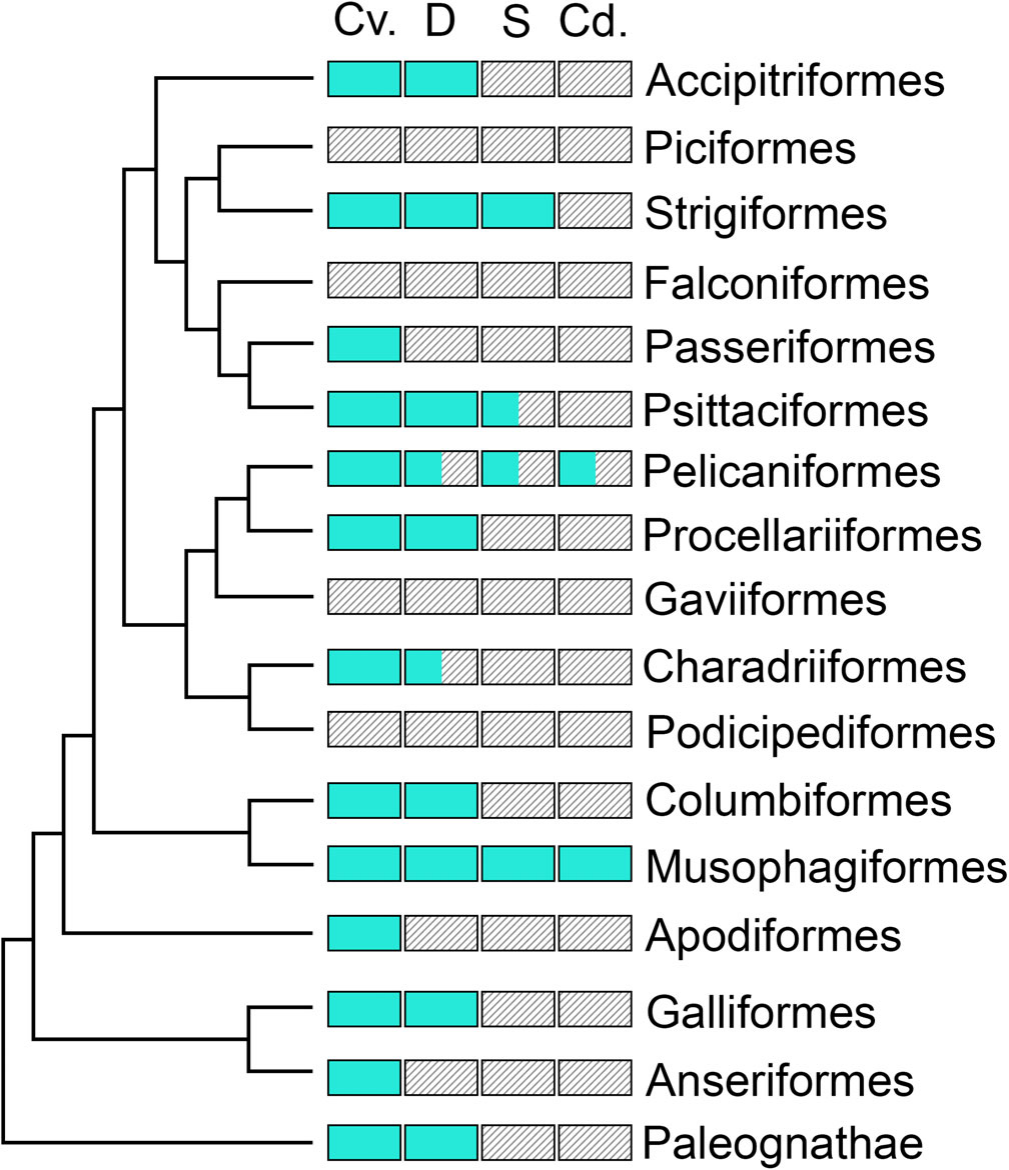
Phylogenetic tree showing clade‐level sampling of this study (topology based on Prum et al., 2015). Diagram indicates the presence (green) or absence (lines) of paramedullary diverticula across vertebral regions. Cv = cervical, D = dorsal, S = sacral, Cd = caudal. Notably, for each clade for which more than one genus was sampled, all genera had identical patterns of presence/absence across vertebral regions

Several other authors have described and illustrated these diverticula in some extant birds, or provided osteological evidence of their presence in fossil taxa. Here we review these prior descriptions and detail the various terms that have been used to refer to these pneumatic diverticula inside the neural canal or otherwise in close contact with the spinal cord (Table 1). However, hereafter and throughout this paper we refer to any diverticula in contact with the spinal cord as “paramedullary diverticula,” for reasons discussed in greater detail below. Where prior authors have used other terms for these structures, those terms are noted. In addition to a literature review, we present the first phylogenetically broad, detailed study of paramedullary diverticula in extant Aves (Figure 2). We have identified four methods for investigating paramedullary diverticula. Each is detailed below.

**TABLE 1:** Summary of the varied nomenclature that has been used to describe paramedullary diverticula and associated respiratory structures

Sappey (1847)	Campana (1875)	Baer (1896)	Müller (1907)	Cover (1953)	O'Connor (2006)	Current paper
*—*	Extra‐spinal pneumatic canal	*Canalis intertransversarius*	*Canalis intertransversarius*	Cervical extension	Lateral vertebral diverticulumm	Intertransverse diverticulum
*—*	*—*	Vertebral part of the clavicular sac	*Diverticulum supravertebrale*	Anterior prolongation	Supravertebral diverticulum	Supravertebral diverticulum
Intra‐spinal pneumatic canal	Intra‐spinal pneumatic canal	Spinal part of the clavicular sac	*Diverticulum supramedullaire*	Intraspinal connection	Supramedullary diverticulum	Paramedullary diverticulum (morphologies ii–iv)
*—*	Pneumatic transverse canal or transverse anastomosing branch	*—*	*—*	—	Anastomosing diverticulum	Paramedullary diverticulum (morphology i)

*Note*: The terminology used to refer to the various diverticula that are present in and around vertebrae is complex, with multiple authors introducing their own new terms. Here, we attempt to summarize, clarify, and simplify this terminology.

### 1.1 Gross anatomical dissection

**FIGURE 3:**
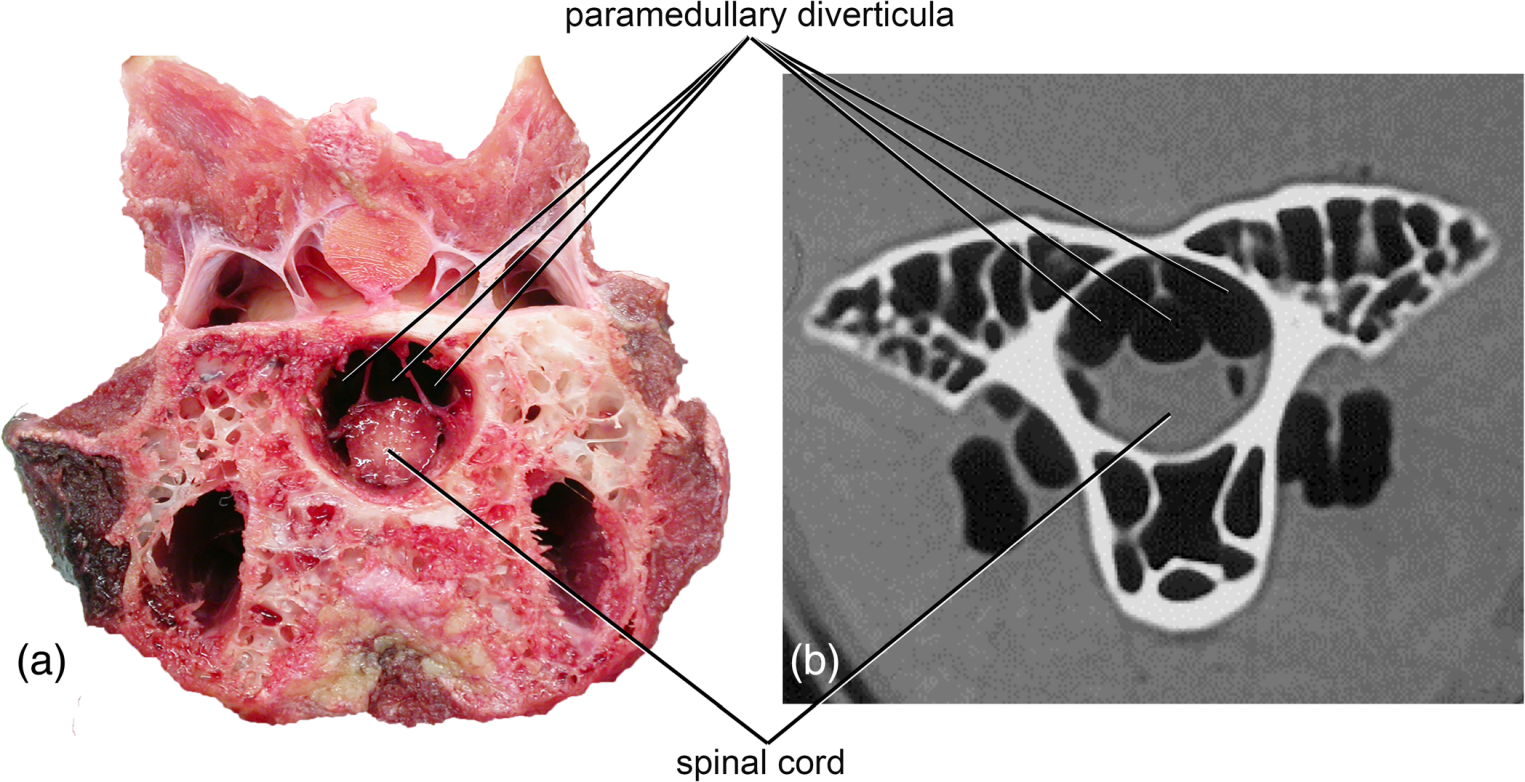
Paramedullary diverticula as seen a dissection and a CT scan. (a) Fresh dissection of an ostrich neck. (b) CT scan of an ostrich neck. In both visualizations, paramedullary diverticula are clearly visible as dark air spaces adjacent to the soft tissue of the spinal cord, contained within the hard bony tissue of the vertebral canal

In many taxa, especially larger‐bodied ones such as turkeys and the ratites, paramedullary diverticula are visible in gross dissection. This is particularly true when the vertebrae are disarticulated from each other or transversely sectioned (see Figure 3a) although we have also observed paramedullary diverticula in articulated vertebrae by dissecting into the space between the zygapophyses of adjacent vertebrae from a dorsal approach.

### 1.2 Physical endocasts

By far, the most‐used method of exploring and documenting the form and extent of the diverticula is to create physical endocasts by injecting all or part of the respiratory system with a casting material, typically latex or resin (e.g., Bezuidenhout et al., 1999; Campana, 1875; Cover, 1953; Müller, 1908; O'Connor, 2006; Stanislaus, 1937). This method allows for very fine diverticula to be preserved and studied, but in long, blind‐ended diverticula such as the paramedullary diverticula, it can be difficult to achieve complete filling of the diverticular network. Furthermore, there is typically no distinct sign of incomplete filling—an incompletely filled diverticulum is simply absent from the cast, the same as a diverticulum that does not typically exist in the taxon under study, or which has not yet developed. Still, physical endocasts remain relatively straightforward and inexpensive to produce.

### 1.3 CTs and digital endocasts

Because paramedullary diverticula are full of air and therefore completely radio‐lucent, they show up quite well in computed tomography (CT) images (Figure 3b). To date, only a handful of images of paramedullary diverticula have been published, and these are mostly “incidental hits” in CT images taken for other purposes—see for example the CT cross‐section of an ostrich neck in Wedel (2003a).

### 1.4 Osteological traces

Documentation of skeletal pneumatization by physical examination of dry bones is by now a well‐established practice (Hogg, 1984). Pneumatic foramina tend to be larger than neurovascular foramina, they lead to internal air spaces that differ in size and geometry from the trabecular spaces in non‐pneumatized bone, and they are often found in association with other pneumatic traces such as tracks and fossae (Britt, 1997; O'Connor, 2006). Although they have been little‐documented to date, pneumatic foramina inside the neural canal are osteological traces of paramedullary diverticula. As with other osteological correlates of pneumaticity, pneumatic foramina inside the neural canal tend to be highly variable among taxa, among individuals within a population or species, and serially along the vertebral column of a single individual.

To our knowledge, no‐one has previously attempted a phylogenetically broad survey of paramedullary diverticula using CT images (or any other medium for that matter). These structures have been previously described in only a handful of genera from six major clades: duck (*Anas platyrhynchos*), Anseriformes (Sappey, 1847); chicken (*Gallus gallus*), Galliformes (Campana, 1875); rock dove (*Columba livia*), Columbiformes (Baer, 1896; Müller, 1908); hummingbirds, Trochilidae (Stanislaus, 1937); turkeys (*Meleagris gallopavo*), Galliformes (Cover, 1953); and ostriches (*Struthio camelus*), Struthioniformes (Bezuidenhout et al., 1999). Of these, only four describe paramedullary diverticula in detail (the studies on *Anas*, *Gallus*, *Columba* and *Meleagris*). In the current study, using CT data, we report on the morphology and variation of supramedullary diverticula in 57 specimens representative of 29 taxa from 17 major avian clades, thereby substantially expanding our knowledge of this little‐known aspect of vertebrate morphology.

## 2 LITERATURE REVIEW

**FIGURE 4:**
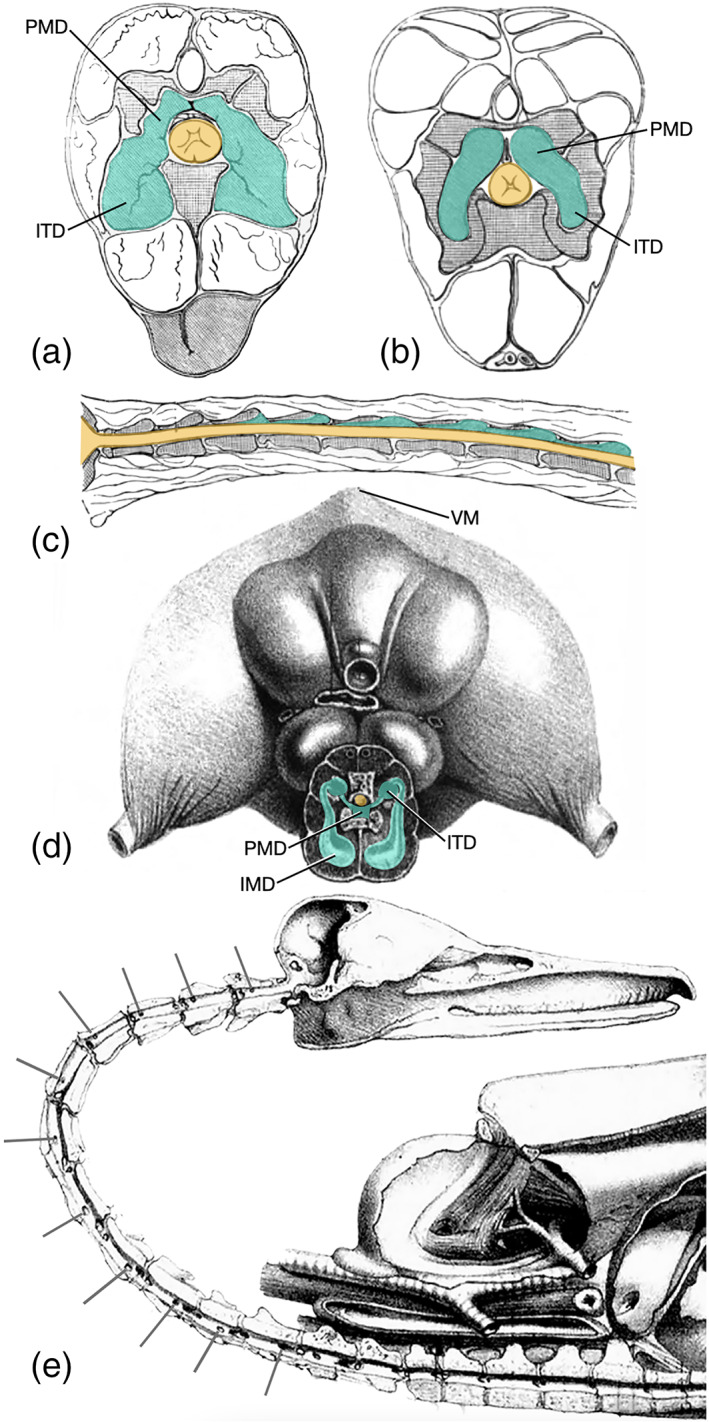
Reproductions of figures from select historical publications showing evidence of paramedullary diverticula; yellow = spinal cord; green = diverticula. Transverse sections through the neck of a domestic chicken modified from Campana (1875) at an intervertebral joint showing the connection between intertransverse diverticula and paramedullary diverticula near an intervertebral joint (a and b). (c) Midsagittal section through the neck of a domestic chicken showing a series of paramedullary diverticula through this vertebral region, also modified from Campana (1875). (d) transverse section through the base of the neck of a duck showing associated air sacs and diverticula, modified from Sappey (1847); note that the bird is depicted lying supine, with the keel of the stenum pointed upward. (e) Midsagittal section through the cranial and cervical skeleton of a duck, showing pneumatic foramina inside of vertebral canals (lines) where paramedullary airways entered the bone, modified from Sappey (1847). IMD, intermuscular diverticulum; ITD, intertransverse diverticulum; PMD, paramedullary diverticulum; VM, ventral midline

The earliest detailed description of paramedullary diverticula that we have been able to find is that of Sappey (1847), who described and illustrated these structures in the duck. Sappey referred to the paramedullary diverticulum as the “canal aérifère intra‐rachidien” or “intra‐spinal air canal.” Specific details in Sappey's description include connections between the paramedullary diverticula and the intertranverse diverticula, and pneumatization of the neural arch from the paramedullary diverticula (Sappey, 1847: plate 3; Figure 4 of current publication).

Campana (1875) described and illustrated paramedullary diverticula in his magisterial description of the respiratory system in the chicken, *Gallus domesticus*, in which he referred to them as “canaux pneumatique intra‐rachidiens,” or “intra‐spinal pneumatic canals.” In this publication, Campana's figures 25–30 all show at least a portion of the paramedullary diverticula (see Figure 4 of the current paper for reproductions of several of these). Campana (1875) described the paramedullary diverticula as originating from the intertransverse diverticula, and he noted numerous anastomoses among the paramedullary diverticula and other pneumatic diverticula adjacent to the vertebrae.

Baer (1896) described and illustrated (in his plate 21) paramedullary diverticula in the pigeon, and their connections to the intertransverse canals. In contrast to Sappey (1847) and Campana (1875), he seems to have considered all diverticula in the neck of the pigeon to be derived from the clavicular sac rather than the cervical sacs; he referred to the paramedullary diverticula as “spinaler theil des clavicularen sackes” or “spinal part of the clavicular sac.” He also described tiny extensions of the paramedullary diverticula that surround the costo‐vertebral articulations in the thoracic region (Baer, 1896, p. 434).

Müller (1908) described and illustrated paramedullary diverticula in the pigeon, *Columba livia*. He established detailed terminology for the various structures he observed. *Canalis intertransversariius* (intertransverse canals) are bilateral tubes running laterally along the vertebral column. In the cervical vertebrae, these pass through the transverse foramina together with the vertebral arteries. *Diverticula supervertebralae* are diverticular expansions of the air sac system on the antero‐dorsal surface of vertebrae. Finally, the terms *diverticulum supramedullaire* (supramedullary diverticula) and *canalis supramedullaris* (supramedullary canal) were reserved for extensions into the vertebral canal which contact the spinal cord (either as separate, paired, or continuous, anastomosing structures, respectively).

**FIGURE 5:**
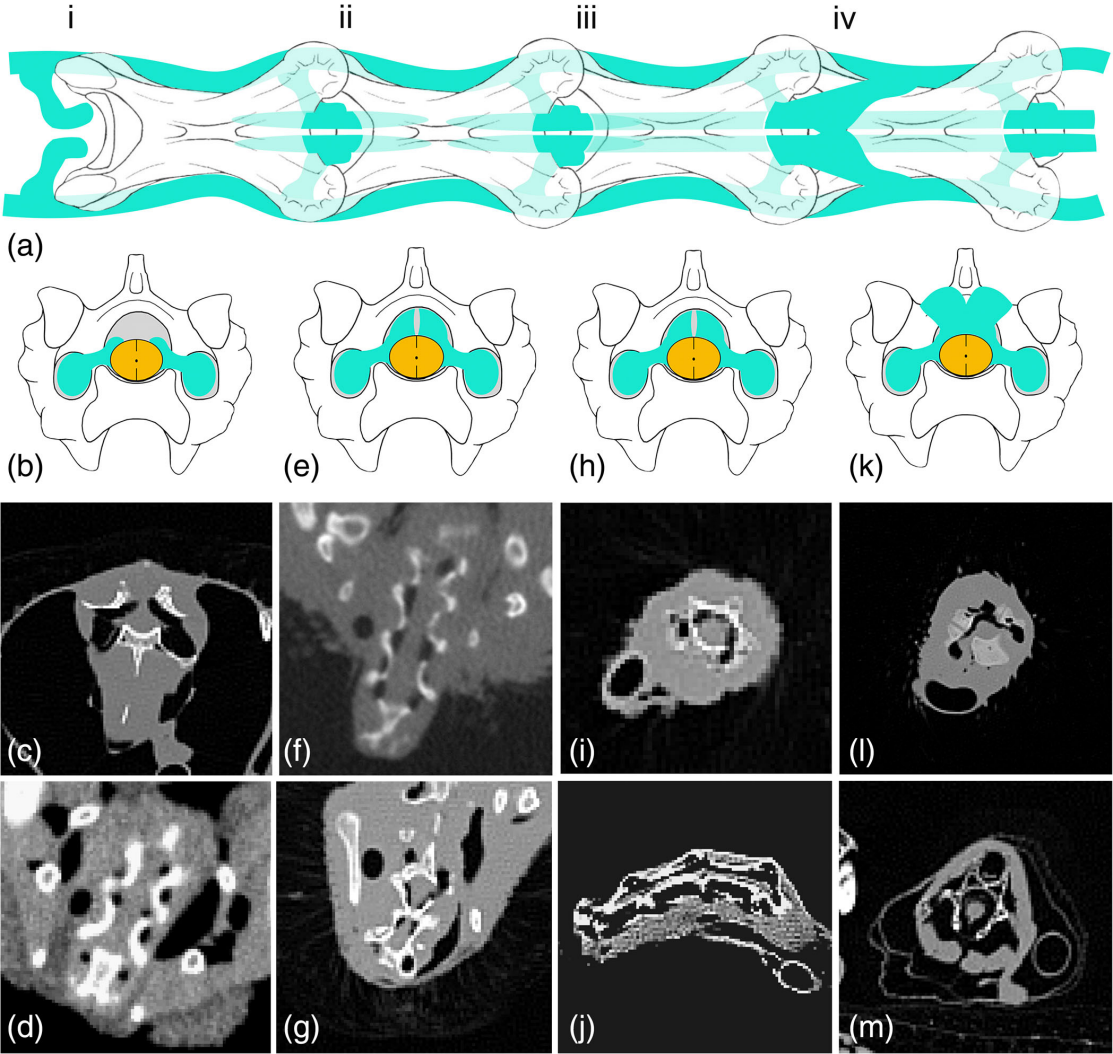
Morphological variation in paramedullary airways; yellow = spinal cord, green = diverticula. The spectrum of variation is discretized into four groups: i, branches of intertransverse diverticula contact spinal cord at intervertebral joints; ii, branches of intertransverse diverticula extend partially into the vertebral canal, but remain discontinuous; iii, paramedullary diverticula form sets of tubes that are continuous through vertebral canals of at least two consecutive vertebrae; iv, continuous paramedullary diverticula anastomose with supravertebral diverticula. Each variant is depicted diagrammatically (a, dorsal view; b, e, h, and k, transverse view) and shown in two CT scans; images in each column correspond to the same morphology. Morphology i: C, cormorant; D, scrub jay. Morphology ii: F, bushtit; G, common murre. Morphology iii: I, red‐tailed hawk; J, black‐crowned night heron. Morphology iv: L and M, pelican

Müller's description of the paramedullary diverticula is concise, detailed, and worth quoting in full (Müller, 1908, p. 377):

The medullary diverticula are given off from the cervical canal just in front of the foramina transversaria. They consist of extravertebral and intravertebral portions. The extravertebral portions are small and simple vesicles. The intravertebral portions, which I name diverticula supramedullaria (fig. 12, DSPM 1; figs. 11 and 12, DSPM 2), enter the medullary canal through the intervertebral foramina, and extend dorsally from the spinal cord. Within the medullary canal they widen out and impinge upon the corresponding diverticula of the opposite side. They partly unite with these as well as with the adjacent diverticula (in front and behind) of the same side, to form a continuous canal, sickle‐shaped in transverse section, and lying above the medulla, the canalis supramedullaris (figs. 3, 4, 5, 7, and 12, MEA). The partial absorption of the walls of these diverticula which leads to the formation of this canal, takes place during the growth of the bird, and posteriorly, near the thorax, where the canal is widest, is usually quite completed in middle‐aged birds. Anteriorly this absorption decreases as the medullary diverticula become smaller, the completely formed supramedullary canal usually extending no farther than the third or fourth cervical vertebra. Anterior to that it is replaced by two rows of isolated diverticula (fig. 12). The posterior end of the supramedullary canal lies near the last cervical vertebra. Occasionally it communicates here with the corresponding canal of the thoracic vertebrae.

Müller (1908, pp. 377–378) went on to describe a similar system in the thoracic vertebrae of the pigeon. He explicitly described the paramedullary diverticula in the thoracic vertebrae as arising from the posterior portions of the cervical air sac, and says that the dorsal ribs are pneumatized by “fine tubules” extending from the paramedullary airways. We will revisit these points below, in the context of more recent descriptive work.

In his description of the lungs of hummingbirds (Trochilidae), Stanislaus (1937, figure 5) illustrated a single midline paramedullary diverticulum arising from paired connections to the cervical air sacs, with lateral extensions that do not seem, from the figure, to form a continuous intertransverse canal on either side. However, the paramedullary and other vertebral diverticula are only illustrated *en passant* and not described in detail, as the paper was focused on the external and internal anatomy of the lungs and bronchi.

Cover (1953, figure 2) illustrated the cervical diverticula in the turkey, *Meleagris gallopavo*, including paramedullary diverticula. Cover did not cite Campana (1875) or Müller (1908) and does not seem to have been aware of their prior work. He used a new and completely different system of nomenclature for the various diverticula in and around the cervical vertebrae (see Table 1). Cover's “cervical extensions” are synonymous with Müller's “intertransverse canals,” as these diverticula represent branches, or extensions, of the cervical air sacs. Cover's “anterior prolongation” is partially analogous to Müller's “supravertebral diverticula.” While Müller's term refers to diverticula on the anterodorsal surface of the vertebra, Cover's describes both this anterodorsal portion as well as a lateral connection with the intertransverse diverticula. This divergence in definitions is likely a consequence of taxonomic variation in diverticular morphology, and the specific taxonomic focus of each study. Müller did not observe a connection between the intertransverse and supravertebral diverticula in the pigeon (the only taxon included in his study). Cover, in contrast, only observed diverticula *with* such a connection in his description of the respiratory system in the wild turkey. Finally, Müller's “*diverticula supramedullaires*” are designated “intraspinal connections” and “dorsal confluence” by Cover (1953, caption of figure 2). Again, this difference hints at previously undescribed variation in diverticular morphology among different taxon, upon which we elaborate in the current study.

In Cover (1953), the in‐text description of the paramedullary diverticula is limited to a single sentence (Cover, 1953, p. 241): “An anastomosing radicle passes from the junction through the vertebral canal dorsal to the spinal cord.” Cover (1953, p. 241) also described extra‐vertebral diverticula of the cervical air sacs passing “caudally along the sides of the vertebrae as far as the fourth coccygeal,” a point that will become important later on.

King (1966) reviewed the then‐existing literature on the cervical diverticula in birds, as part of a larger paper on the structure and function of the lungs and air sacs. He consistently referred to paramedullary diverticula as “dorsal tubes,” although this seems to have been deliberately informal and not an attempt to establish novel anatomical terminology. His descriptions of the paramedullary diverticula generally follow those of Müller (1908) and Cover (1953). In particular, King (1966) followed Müller (1908) in describing paramedullary diverticula in the postcervical vertebral column as having originated from the cervical air sacs—a point disputed by later authors.

Bezuidenhout et al. (1999, p. 324) described the paramedullary diverticula in the ostrich (*Struthio camelus*) as follows:

The cranial vertebral diverticula were tubular structures that accompanied the vertebral blood vessels through the transverse canal of the cervical vertebrae. They extended to the level of the axis (C2). Along the way they gave off supravertebral diverticula which lay around the articular processes and supramedullary diverticula that passed through the intervertebral foramina to form a continuous tube dorsally to the spinal cord.

The most recent detailed description of the paramedullary diverticula is that of O'Connor (2006), who used a combination of anglicized Müller terminology in together with original jargon, sometimes renaming Müller's structures and in other cases naming structures not previously described. O'Connor applied the term “lateral vertebral diverticulum” in place of Müller's “*canalis intertransversariius*,” but adapts his “*diverticulum supravertebrale*” and “*diverticulum supramedullaire*” as “supravertebral diverticula” and “supramedullary diverticula” respectively. He also coined a term for the diverticula that connect the supramedullary and intertransverse canals at intervertebral joints, referring to them as “anastomosing diverticula.”

O'Connor (2006, table 1) defined the paramedullary diverticula (supramedullary diverticula or “SMDv” of his usage) as follows (table 1 in O'Connor, 2006): “longitudinal system variably occupying the extradural space within the vertebral canal. The SMDv is made up of contributions from (1) the cervical air sac, (2) pulmonary diverticula of the lung, and (3) perirenal diverticula of the abdominal air sac.” O'Connor (2006) went on to provide detailed descriptions of the paramedullary diverticula derived from the cervical air sacs (pp. 1210–1211) and the abdominal air sacs (pp. 1211–1212), making the following key points:

- Paramedullary diverticula may form a single dorsal tube, two or more parallel tubes, or a jacket that completely surrounds the spinal cord, which O'Connor (2006, p. 1210) refers to as a “peridural diverticulum.”
- The paramedullary diverticula are the source of the supravertebral diverticula; this is contra Cover [1953], who described the paramedullary diverticulum as arising from the supravertebral diverticulum (“anterior prolongation” of his usage).
- No diverticula of the cervical air sacs, including the cervical paramedullary diverticula, extend farther caudally than the mid‐thoracic region. Paramedullary diverticula in the thoracic, synsacral, and caudal regions of the vertebral column arise from the lungs or abdominal air sacs, although they may anastomose with diverticula of the cervical air sacs in the mid‐thoracic region. This is contra Cover (1953) and King (1966), both of whom explicitly described postcervical vertebral diverticula as having arisen from the cervical air sacs.
- If paramedullary diverticula derived from the cervical and abdominal air sacs anastomose, “cranial (cervical air sac diverticula) and caudal (abdominal air sac diverticula) components of the air sac system can communicate with one another via the vertebral canal” (p. 1211).
- Dorsal ribs may be pneumatized by lateral vertebral diverticula of the cervical air sacs, or by pulmonary diverticula of the lung itself (p. 1211), but not by the paramedullary diverticula (contra Müller, 1908).

From the foregoing descriptions and discussions, several points remain unresolved, which are discussed below.

### 2.1 Do paramedullary diverticula of the cervical air sac extend into the thoracic region, or to other more posterior regions of the vertebral column?

Müller (1908) actually described two different sets of paramedullary diverticula originating from the cervical air sacs in the pigeon. The first arises at each intervertebral joint in the cervical column as a bilateral, medial extension of the intertransverse diverticulum. This system terminates “near the last cervical vertebra” (p. 377).

Müller (1908: pp. 377–378) described the second system as follows:

From the distal end of the pars ovalis [of the cervical air sac] of either side a ventrally flattened tube arises. This passes between the vertebral muscles and through the intervertebral foramen in front of the first thoracic vertebra into the spinal canal, where it unites with the corresponding tubule from the opposite side, both together forming a duct similar to the canalis supramedullaris. This duct extends backward but does not reach the last thoracic vertebra. It is very variable, and sends fine branches into the vertebrae and the ribs.

The second system is described as having an inside‐to‐outside developmental sequence, with the paramedullary diverticula giving rise to extravertebral diverticula and pneumatizing the dorsal ribs. In contrast, O'Connor (2006) argued that the dorsal ribs were pneumatized by lateral vertebral diverticula of the cervical air sacs, or by pulmonary diverticula of the lungs, and that the paramedullary diverticula in the thoracic region could arise from the pulmonary diverticula or as anterior extensions from the abdominal air sacs. It is interesting to note that Müller (1908, p. 378) allowed that, “It has sometimes seemed to me that the ribs were pneumatized directly from the lungs,” which is consistent with the findings of O'Connor (2006). We also note that in the absence of developmental information, it is impossible to tell whether an anastomosing system developed from outside‐to‐inside or vice versa. Possibly the paramedullary diverticula in the thoracic region of the pigeon do in fact arise from extra‐vertebral diverticula of the lungs, as described by O'Connor (2006), and Müller (1908) simply got the arrow of developmental history backwards.

There is also the contention of Cover (1953) and later secondary sources (e.g., King, 1966, 1975) that extra‐vertebral diverticula of the cervical air sac extend back as far as the tail. This possibility is strongly contradicted by O'Connor (2006), who found that no cervical diverticula of any form persisted farther posteriorly than the mid‐thoracic region. This may be another case of ontogenetic confusion, in which the anastomosis of several, originally independent sets of vertebral diverticula give rise to a continuous airway, which earlier authors mistakenly attributed entirely to the cervical air sacs. Finally, we note that Schachner et al. (2021) report that the primary source of the various vertebral diverticula found in the cervical region is in fact the cranial portion of the lungs, rather than the cervical air sacs as described in other birds (though the authors note that connections between the cervical air sacs and vertebral diverticula are still very likely). These various findings indicate that ontogenetic data and greater interspecific sampling are needed to resolve these issues.

### 2.2 Do paramedullary diverticula more typically form as a single tube, paired tubes, or multiple tubes, and how does this vary among regions of the vertebral column and among taxa?

This question probably does not represent differences of fact or opinion among previous authors, but rather the actual diversity of morphology of paramedullary diverticula among different taxa of birds. Müller (1908) described paired tubes in the cervical region of the pigeon, and a single unpaired tube in the thoracic region. Stanislaus (1937) and Cover (1953) illustrated unpaired tubes in the necks of hummingbirds and turkeys, respectively. O'Connor (2006) described the cervical paramedullary diverticula as forming a single tube in some taxa (e.g., ducks), parallel tubes in others (e.g., ostriches), and a complete jacket enclosing the spinal cord in still others (e.g., storks and pelicans). We should therefore be alert to variation in the form and extent of the paramedullary diverticula along the vertebral column in individual birds, and also to pronounced variations among taxa.

### 2.3 Museum acronyms

Acronyms are as follows: LACM, Los Angeles County Museum, Los Angeles, CA, USA; MVZ, Museum of Vertebrate Zoology, University of California, Berkeley, CA, USA.

## 3 MATERIALS AND METHODS

### 3.1 Nomenclature

Though comparatively few previous publications report on paramedullary (formerly “supramedullary”) diverticula, the nomenclature created to describe diverticular anastomoses in and around avian vertebrae has a complex history and is at times both convoluted and redundant. A summary of the history of terms used, along with a clarification of nomenclature applied in this paper, can be found in Table 1. In the current study, we primarily use anglicized versions of Müller (1908). We also retain usage of Cover's (1953) “anterior prolongation,” adapting the term to apply to the diverticula connecting the supravertebral and intertransverse diverticula.

However, based on the results of the current study, we adopt a new term in place of “supramedullary diverticula” or “intraspinal connections.” As described in detail below, we document high levels of variation in the morphology and arrangement of these structures, and find that they are neither exclusively “supra” relative to the spinal cord, nor are they necessarily restricted to the vertebral canal as implied by the descriptor “intraspinal.” In his expansive paper on the avian air sac system, O'Connor (2006) also mentions such variation, even suggesting that in some cases “peridural” is a more apt descriptor of these diverticula. Based on our detailed observations, we suggest the name of these structures be changed to the more general “paramedullary diverticula” and refer to them as such throughout this work. Because paramedullary diverticula exhibit such a varied gradation of morphologies, we do not apply O'Connor's term “anastomosing diverticula”; evidence presented here instead suggests that these are merely one morphological variant of paramedullary diverticula.

### 3.2 Data collection

Naturally deceased specimens were received as donations from the OK Corral Ostrich Farm in Oro Grande, CA (the source of the ostriches); Avian Resources in San Dimas, CA (the source of all other exotic taxa not native to California); and the Lindsay Wildlife Museum in Walnut Creek, CA, and the Society for Prevention of Animal Abuse in Monterey Co., CA (the source of all California native taxa). This study is intended as a preliminary general survey of paramedullary airways, and includes all available specimens regardless of age. Some taxa are represented by partial growth series, others only by adults or only chicks. It will be the focus of future work to explore the ontogeny of these structures in greater detail. Precise ages of most individuals at time of death is unknown in most cases; however, specimens were classified in the following qualitative growth stages based on identifications made by the wildlife hospitals, body size, and general external morphology (primarily the condition of the feathers): neonate, downy chick, pin‐feathered chick, prefledgling chick, fledgling chick, sub‐adult, and adult (Table 2).

**TABLE 2:** Summary of morphological variation of paramedullary diverticula by individual taxon, and by larger clade

Clade	Taxon	PMD morphologies present	PMD orientation to spinal cord	Museum number	Ontogenetic stage
Accipitriformes	*Buteo jamaicensis*	ii	Two tubes, dorsal	MVZ 190852 MVZ 190855	Adult Adult
	*Cathartes aura*	ii	Two tubes, dorsal	LWH 2008‐0151	Adult
Apodiformes	*Calypte anna*	ii iii iv	One tube, dorsal	MVZ 190804 MVZ 190813 MVZ 190807	Adult Subadult Adult
Anseriformes	*Anas platyrhynchos*	i ii	Two tubes, dorsal	MVZ 190735 MVZ 190736 MVZ 190740	Downy chick Downy chick Downy chick
Charadriiformes	*Larus occidentalis*	ii iii	One tube, dorsal and lateral	MVZ 190829 LWH 2009‐0051	Adult Adult
	*Uria aalge*	ii iii	Two tubes, dorsal One tube, dorsolateral	MVZ 190818	Adult
Columbiformes	*Zenaida macroura*	i ii	Two tubes, dorsal	MVZ 190779 MVZ 190791 MVZ 190787	Fledgling Fledgling Fledging
Falconiformes	*Falco sparverius*	Absent	Absent	MVZ 190891 MVZ1 90,886	Fledgling Prefledgling
Galliformes	*Meleagris gallopavo*	ii iii iv	Two tubes, dorsal	MVZ 190763	Adult
Gaviiformes	*Gavia pacifica*	—	—	SVPCA 2012‐0594 SVPCA 2012‐0895	Adult Adult
	*Gavia adamsii*	—	—	MVZ 190834	Adult
	*Gavia immer*	—	—	PLC 607	Adult
Musophagiformes	*Musophaga violacea*	ii iii	Two tubes, ventral One tube, ventrolateral Two tubes, lateral Two tubes, dorsal One tube, dorsal Four tubes, dorsolateral Encircling	JAA 308	Adult
Passeriformes	*Aphelocoma californica*	i ii	Two tubes, dorsal	MVZ 190924 MVZ 190921	Adult Prefledgling
	*Melozone crisallis*	iii iv	Two tubes, dorsal	MVZ 191010 MVZ 191011	Fledgling Fledgling
	*Psaltriparus minimus*	ii iii	One tube, dorsal	LWH 2011‐1420 MVZ 190955 MVZ 190953 LWH 2011‐1268 LWH 2011‐1269 MVZ 190954	Fledgling Fledgling Pin‐feathered chick Pin‐feathered chick Pin‐feathered chick Pin‐feathered chick
	*Spinus psaltria*	ii iii	Two tubes, dorsal	MVZ 191000 MVZ 191001	Fledgling Prefledgling
Pelicaniformes	*Nycticorax nycticorax*	ii iii	Two tubes, dorsal One tube, dorsal	MVZ 190846 LWH 2010‐0006	Fledgling Adult
	*Pelecanus occidentalis*	iv	Encircling Two tubes, dorsal	MVZ 190839 MVZ 190841	Adult Adult
	*Phalacrocorax penicillatus*	i ii iii	Two tubes, dorsal	MVZ 190837 MVZ 190838	Adult Adult
Piciformes	*Dryobates nuttallii*	—	—	LWH 2011‐2064	Adult
	*Melanerpes formicivorus*	—	—	LWH 2011‐2880	Adult
Podicipediformes	*Aechmorphus occidentalis*	—	—	MVZ 190769 MVZ 190770	Adult Adult
	*Aechmorphus clarkii*	—	—	MVZ 190767 MVZ 190768	Adult Adult
Procellariiformes	*Puffinus griseus*	ii iii	One tube, dorsolateral	SPCA 2012‐0543 SPCA 2012‐0859 SPCA 2012‐543	Adult Adult Adult
Psittaciformes	*Eclectus roratus*	ii iii	Encircling Two tubes, dorsal One tube, dorsal	JAA 307	Adult
	*Pyrrhura molinae*	ii iii	Two tubes, dorsal	MVZ 190908 MVZ 190907	Subadult Subadult
Strigiformes	*Bubo virginianus*	ii	Two tubes, dorsal	MVZ 190883 MVZ 190882 MVZ 190881	Adult Adult Pin‐feathered chick
Struthioniformes	*Struthio camelus*	ii iii iv	Two tubes, dorsal One tube, dorsolateral Three tubes, dorsolateral	MVZ 190732	Downy chick

*Note*: Notably, in clades for which more than one genus was sampled the presence or absence of paramedullary diverticula is ubiquitous. Roman numerals refer to the four main morphological variants of paramedullary diverticula: i, branches of intertransverse diverticula contact spinal cord at intervertebral joints; ii, branches of intertransverse diverticula extend partially into the vertebral canal, but remain discontinuous; iii, paramedullary diverticula form sets of tubes that are continuous through vertebral canals of at least two consecutive vertebrae; iv, continuous paramedullary diverticula anastomose with supravertebral diverticula. The order of these as reported in this table is simply numerical, and not necessarily reflective of their actual anteroposterior order of occurrence. Oftentimes multiple morphologies are present in a single individual or taxon, see text for discussion. For some specimens, a formal specimen number has not yet been assigned, in which case the field number is reported.

Abbreviation: PMD, paramedullary diverticula.

MicroCT scanning of small‐bodied specimens (approximately 15 cm or less in length) occurred at the Center for Molecular and Genomic Imaging (CMGI) at the University of California, Davis, on a Zeiss Xradia microXCT‐200 (slice thickness = 48 μm) and the Oregon Health Sciences University in Portland, Oregon on a Perkin Elmer Quantum FX microCT scanner (slice thickness = 100 μm). Scans of larger specimens were acquired at the University of Utah Medical Center Research Park in Salt Lake City, UT, on a 164 slice Siemens single source medical CT scanner (slice thickness = 0.6–1 mm). We examined two‐dimensional slices from these conventional CT and microCT scans of dead specimens in the program ImageJ (v1.53g). Most specimens have been skeletonized and are reposited in this museum (several were dissected for other study and disposed of).

Osteological specimens in the Ornithology collections of the Natural History Museum of Los Angeles County were also observed in searching for further examples of osteological correlates of paramedullary airways.

## 4 RESULTS

Paramedullary diverticula are common, but not omnipresent, among extant birds. While most groups in our dataset possess these diverticula (19 genera), they were completely absent in others (5 genera) (Table 2). Notably, for all clades for which we were able to sample more than one genus (Accipitriformes, Charadriiformes, Passeriformes, Pelicaniformes, and Piciformes) members are ubiquitously characterized by either the presence or absence of diverticula in contact with the spinal cord. Thus, based on current sampling, this character has strong, unequivocal phylogenetic signal.

Much variation in morphology exists not only when comparing taxa or individual specimens, but often within the vertebral column of a single individual. We describe the following four common morphologies (Figure 5): (i), intertransverse diverticula branch and contact spinal cord at intervertebral joints; (ii), branching of intertransverse diverticula at intervertebral joints extends partially into the vertebral canal, but is discontinuous with diverticula of the following joint; (iii), supramedullary diverticula form sets of tubes that are continuous through vertebral canals of at least two consecutive vertebrae; (iv), continuous diverticula through vertebral canal anastomose with supravertebral diverticula. It is important to note, first, that these discrete descriptions in fact represent a continuum of morphologies; second, that within a single individual, combinations at least two of these morphologies were often observed; and third, that other combinations exist (e.g., continuous paramedullary diverticula connected only with supravertebral diverticula), though these are rarer.

**FIGURE 6:**
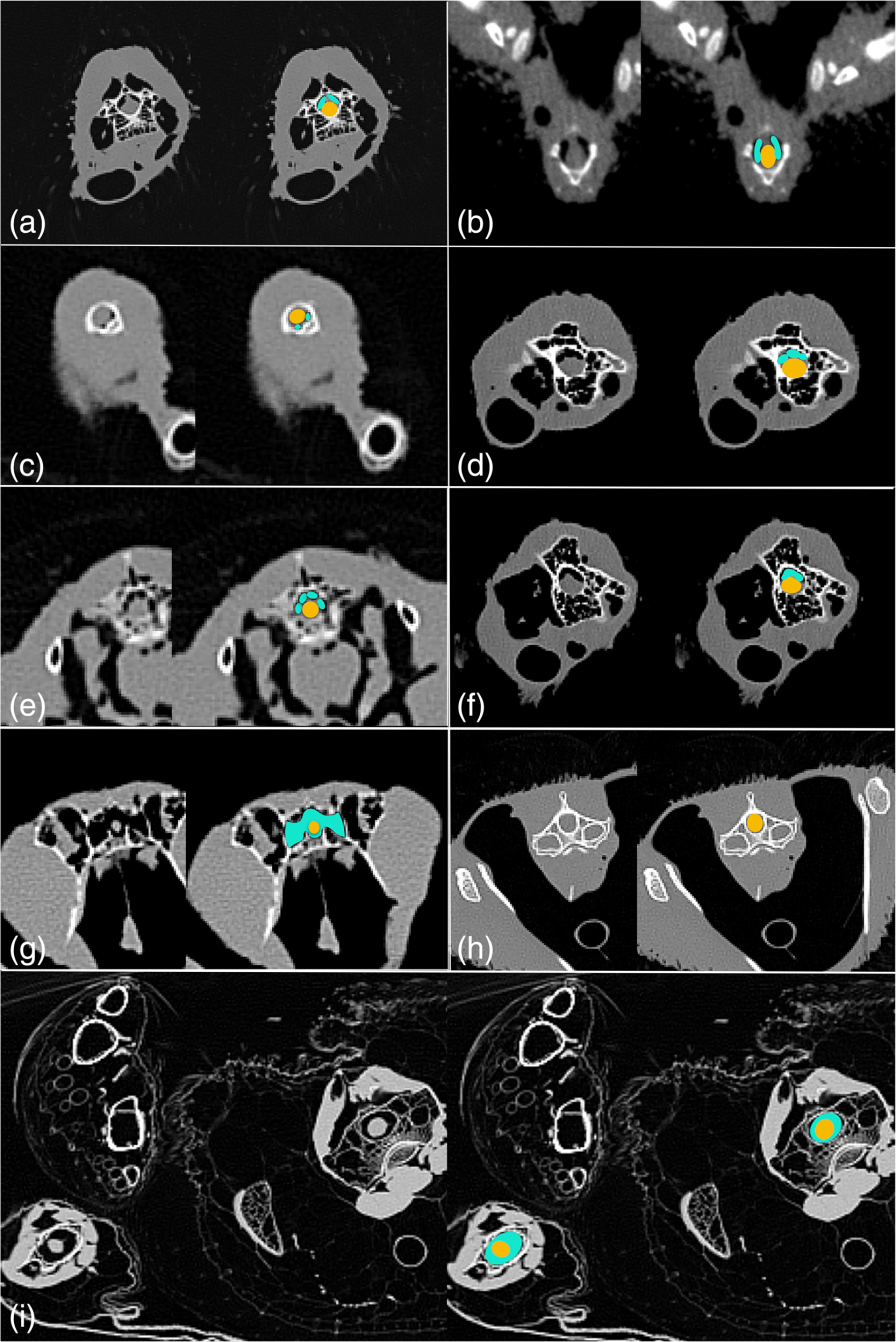
Observed variation in the shape, arrangement, and orientation of paramedullary diverticula relative to the spinal cord; yellow = spinal cord, green = diverticula. (a) Paired diverticula dorsal to spinal cord in an ostrich. (b) Paired diverticula lateral to spinal cord in a bushtit. (c) Paired diverticula ventral to spinal cord in a violet turaco. (d) Three diverticula dorsal to spinal cord in an ostrich. (e) Four diverticula dorsal to spinal cord in an eclectus parrot. (f) Single, c‐shaped diverticulum dorsal to spinal cord in an ostrich. (g) Diverticula completely surrounding spinal cord and pneumatizing vertebra in a violet turaco. (h) No paramedullary diverticula present in a Pacific loon. (i) Diverticula completely surrounding spinal cord in a pelican

Additionally, we observed notable variation in the shape, arrangement, and orientation of diverticula relative to the spinal cord (Figure 6). One common morphology was a pair of tubes within the vertebral canal. Indeed, paired paramedullary diverticula were observed in at least some portion of the vertebral column in every taxon where such structures were present, except the hummingbird, pelican, Western gull, and black‐crowned night heron. These paired paramedullary diverticula are in most instances located dorsal to the spinal cord, but were observed lateral to the cord in the great‐horned owl, bushtit, and Western scrub jay, and ventral to the cord in the violet turaco.

Not uncommonly, we observed three tubes dorsal to the spinal cord, rather than two. Sometimes the diverticula merge into one, single C‐shaped tube in contact with the spinal cord, often dorsally but occasionally latero‐dorsal (and therefore asymmetrical). We note, though, that in at least in some examples where this was observed, we believe there were in fact two or three tubes closely adpressed together, separated by layers of epithelium so thin they were not visible on the CT scans. Perhaps the most striking morphology was seen in pelicans, in which the diverticula completely surround the spinal cord (as also reported by O'Connor, 2006) through the entire vertebral column (Figure 6i), except the anterior and mid‐sacrals.

Not only do these diverticula enter the vertebral canal, but frequently they also pneumatize the bone of the vertebral arch and body from within the canal. This was seen in the violet turaco, eclectus parrot, and pelican. In the pelican, the paramedullary diverticula enter the vertebral body via a foramen in the floor of the canal, and expand such that a section of the body of the vertebra was highly pneumatized and mainly consisted of air in a jacket of thin‐walled bone (Figure 6g).

### 4.1 Descriptions of paramedullary diverticula by clade

#### 4.1.1 Accipitriformes

*Buteo jamaicensis* (red‐tailed hawk): In the cervical vertebrae of red‐tailed hawks paramedullary diverticula are present as paired tubes dorsal to the spinal cord, within the vertebral canal but discontinuous in consecutive vertebrae (morphology ii; Figure 5). These structures persist into the dorsal vertebrae, where they remain paired, discontinuous and dorsal to the spinal cord relative to most vertebrae in this region. They are only variably present in the dorsal vertebrae, and completely absent in the synsacrum. One individual examined had a minute, continuous, single canal dorsal to the spinal cord in the free caudal vertebrae. The other specimen lacked diverticula in the caudal region.

*Cathartes aura* (turkey vulture): Paramedullary diverticula are present in the cervical region as discontinuous, short tubes in the turkey vulture (morphology ii; Figure 5). They are paired and occur dorsal to the spinal cord. In the dorsal vertebrae they become more reduced, losing the posterior expansions into the vertebral canal but maintaining anterior expansions. They are present in the anterior dorsals but absent in the posterior section of this region. Paramedullary diverticula are absent in the synsacrum and caudal vertebrae.

#### 4.1.2 Apodiformes

*Calypte anna* (Anna's hummingbird): In Anna's hummingbirds, cervical vertebrae exhibit variably continuous (morphology iii) and discontinuous (morphology ii) paramedullary diverticula, which frequently anastomose with supravertebral diverticula (morphology iv). These occur as a single, kidney‐shaped tube dorsal to the spinal cord. Though hummingbird body size is very tiny, diverticula within the vertebral canal in this region are substantial, occupying on average about one third of the area of the space. Such diverticula are absent in all other vertebral regions.

#### 4.1.3 Anseriformes

*Anas platyrhynchos* (mallard duck): In this study, mallard ducks were represented only by downy chicks; however, paramedullary diverticula are already present and well‐developed in the cervical region where they appear as paired tubes dorsal to the spinal cord. These structures are substantial, occupying about one third of the area of the vertebral canal. In anterior cervicals, when present, they are discontinuous and exist only at intervertebral joints (morphology i). In the mid‐cervicals, the diverticula are also continuous through consecutive vertebrae. They are absent in all other vertebral regions.

#### 4.1.4 Charadriiformes

*Larus occidentalis* (Western gull): In the cervical region of Western gulls, paramedullary diverticula occur as a single, large, C‐shaped tube that encases the spinal cord dorsally and laterally. In one observed individual, they form continuous tubes from vertebra to vertebra in the anterior cervicals. In a second individual they were absent. In the posterior cervicals of both individuals, they become discontinuous, but still enter the vertebral canal (morphology ii). In the dorsal region paramedullary diverticula are small in the anterior vertebrae and absent in the posterior vertebrae. They are completely absent in the synsacrum and caudal region.

*Uria aalge* (common murre): In common murres, paramedullary diverticula are large (one fourth to one third of vertebral canal area) and exhibit a range of morphologies across the cervical vertebrae. Anteriorly, they arise as paired tubes dorsal to the spinal cord. In the mid‐cervicals, these grade into a single C‐shaped canal continuous through consecutive vertebrae (morphology iii). This in turn diminishes abruptly, and the posterior cervicals completely lack such diverticula. These structures are also absent in all more posterior vertebrae.

#### 4.1.5 Columbiformes

*Zenaida macroura* (mourning dove): In the cervical region of mourning doves, substantial paramedullary diverticula were observed. While absent in the anterior vertebrae, they appear in mid‐ and posterior cervicals as paired tubes dorsal to the cord and oblong in shape (in transverse view). They first appear only at intervertebral joints (morphology i) but soon expand to invade the vertebral canal (morphology ii). Sometimes they become large enough to contact along the dorsal midline, forming a C‐shaped canal. In the anterior dorsal vertebrae, paramedullary diverticula are paired and in contact with the cord only at intervertebral joints (morphology i). In the posterior dorsal vertebrae very large paramedullary diverticula (approximately two‐thirds of area of vertebral canal) were observed as a single, C‐shaped tube dorsal to the spinal cord continuous through consecutive vertebrae (morphology i).

#### 4.1.6 Falconiformes

*Falco sparverius* (American kestrel): Paramedullary diverticula are absent in American kestrels.

#### 4.1.7 Galliformes

*Meleagris gallopavo* (wild turkey): The paramedullary diverticula in the cervical region of the wild turkey are paired and discontinuous through vertebral bodies. In the more posterior vertebrae of this region, they merge with the supravertebral diverticula at intervertebral joints but remain discontinuous, exhibiting a combination of morphologies ii and iv. In the dorsal region, paramedullary diverticula become larger and merge to form a C‐shaped canal at intervertebral joints, connected directly to the cervical air sacs. These structures invade the canal anteriorly, but only slightly; inside of the canal the size is highly reduced. Small connections to supravertebral diverticula were also observed. In the mid‐dorsals, paramedullary diverticula decrease in size overall and appear as thin, C‐shaped tubes at intervertebral joints, connected directly to large, bilateral diverticula emanating from the lungs directly. Going into the vertebral canal they appear as paired, squished tubes and are discontinuous through consecutive vertebrae (morphology iii). In posterior dorsals these structures become highly reduced, and are absent in the sacral and caudal vertebrae.

#### 4.1.8 Gaviiformes

*Gavia pacifica* (Pacific loon): Paramedullary diverticula are absent in Pacific loons.

*Gavia immer* (common loon): Paramedullary diverticula are absent in common loons.

*Gavia adamsii* (yellow‐billed loon): Paramedullary diverticula are absent in yellow‐billed loons.

#### 4.1.9 Musophagiformes

*Musophaga violacea* (violet turaco): Paramedullary diverticula in the violet turaco exhibit a range of unusual and elaborate morphologies, and are present in all four vertebral regions. In the cervicals, they are paired, ventral to the spinal cord, and very tiny in the atlas. These merge to form a single, thin, C‐shaped tube ventral to the cord in the axis. In other cervical vertebrae, paramedullary diverticula are paired, small, and lateroventral, but shift to a more lateral position and become continuous in the posterior neck (morphology iii).

In the dorsal region, these diverticula become larger and discontinuous, persisting as paired tubes now dorsal to the spinal cord. In one vertebra, we observed a unique morphology of two bilateral pairs of small, circular diverticula, forming a total of four separate tubes. These subsequently merge to form a single, C‐shaped continuous tube dorsal to the spinal cord in the mid‐dorsals, and become discontinuous and paired again in posterior dorsals. In the mid‐ and posterior dorsal vertebra, paramedullary diverticula connect directly to the lungs.

In the synsacrum, diverticula are not continuous with those from the dorsal vertebrae. Anteriorly, a single, unpaired diverticulum is the first structure apparent, though paired tubes rapidly appear. These canals are flattened and appear both ventral and dorsal to the cord. Moving posteriorly, they eventually expand to meet laterally, fully jacketing the last sacral vertebra. Paramedullary diverticula in this region are connected to perirenal diverticula.

The spinal cord is bounded dorsally by a single, very large tube through most of the free caudals, and posteriorly becomes fully jacketed. This morphology persists into the pygostyle, where the spinal cord is very small and completely surrounded by paramedullary diverticula. These also appear to originate from the perirenal diverticula.

#### 4.1.10 Passeriformes

*Aphelocoma californica* (scrub jay): In scrub jays, paramedullary diverticula were only observed in the cervical vertebrae. Here, they are paired tubes lateral to the spinal cord present in posterior cervicals only. They are discontinuous and variably enter the vertebral canal to a greater or lesser degree (ranging between morphologies i and ii). In the single adult observed, the canals were uniformly small. In a prefledgling individual also observed, they become much larger moving posteriorly in this region, hinting at possible variation through ontogeny.

*Melozone crisallis* (California towhee): Of the two California towhees included in this study, one had paramedullary diverticula only in the cervical vertebrae. In the other, they were present in the cervicals and first two dorsal vertebrae. In the cervical region, diverticula are paired and continuous anteriorly, expanding to become very large at intervertebral joints and appearing as smaller, squished tubes inside of the canal. Notably, in the anterior cervicals, paramedullary diverticula primarily connect to the supravertebral diverticula and only merge with small, intertransverse diverticula mid‐way through the neck (a mosaic combination of morphologies iii and iv). Eventually, in the posterior cervicals, the intertransverse diverticula become dominant and invade the vertebral canal increasingly less, until paramedullary diverticula are absent in all more posterior vertebrae.

*Psaltriparus minimus* (bushtit): Six bushtits were included in this study. Of these, four were pin‐feathered chicks (very immature), all of which lacked paramedullary diverticula. However, two older individuals (fledglings) had paramedullary diverticula in the cervical vertebrae. These structures do not arise until mid‐neck, where a single, asymmetrical tube first appears before becoming bilaterally paired. In this taxon, the canals are sizeable, occupying about one third of the area of the vertebral canal. They invade the canal and are variably continuous between consecutive vertebrae (a combination of morphologies ii and iii).

*Spinus psaltria* (lesser goldfinch): Paramedullary diverticula in the lesser goldfinch are minute and only present in some cervical vertebrae. They are absent in all other vertebral regions. They were observed as small, paired tubes alternately continuous and discontinuous between consecutive vertebrae (morphologies ii and iii) in the posterior‐most cervicals only.

#### 4.1.11 Pelicaniformes

*Nycticorax nycticorax* (black‐crowned night heron): In the black‐crowned night heron, paramedullary diverticula are present only in the cervical vertebrae and absent in all other regions. General diverticular morphology around these vertebrae was very elaborate, with particularly prominent intertransverse canals and large paramedullary diverticula. In anterior and mid‐cervicals, the paramedullary diverticula invade the vertebral canal and grade between serially continuous and discontinuous (morphologies ii and iii). In posterior cervicals, they appear as a single C‐shaped tube dorsal to the spinal cord and discontinuous between consecutive vertebrae (morphology ii). There were no notable differences between the fledgling chick and adult.

*Pelecanus occidentalis* (brown pelican): Paramedullary diverticula in the brown pelican are large, elaborate, and present in most of the vertebral column. In the cervical vertebrae, these structures first appear in the anterior cervicals posterior to the atlas and axis. They completely encircle the spinal cord, merge with both the intertransverse and supravertebral diverticula, and are serially continuous throughout the vertebrae of this region (morphology iv). In the dorsal vertebrae, this morphology persists. Within the vertebral canal, these diverticula pneumatize the vertebral body via foramina in the floor of the canal in three consecutive vertebrae. Paramedullary diverticula are absent in posterior dorsals and through most of the synsacrum. They were only observed in the posterior‐most sacral vertebrae, as miniscule, paired tubes. In the free caudal vertebrae, they once again expand to nearly or fully encircle the spinal cord (varying between O‐ and U‐shaped).

*Phalacrocorax penicillatus* (Brandt's cormorant): Paramedullary diverticula are restricted to anterior vertebral regions in Brandt's cormorant. They are present in the cervical vertebrae, and occasionally extend into the anterior‐most dorsal vertebrae. In the cervical region, they first appear as small, paired tubes dorsal to the cord in the mid‐cervicals. These quickly expand to become C‐shaped and extend much further into vertebral canals. However, the size of the canals changes dramatically as they extend, becoming much smaller at the mid‐point of each vertebra. Thus, they appear as barely continuous, connecting to each other at their point of smallest volume (morphology ii grades into morphology iii). In the posterior cervicals, they once again become small, paired, and discontinuous. In one individual observed, they ceased at the end of the cervical series. In another, they appeared as small, paired, and present only at intervertebral joints in the anterior‐most dorsals.

#### 4.1.12 Piciformes

*Dryobates nuttallii* (Nuttall's woodpecker): Paramedullary diverticula are absent in Nuttall's woodpeckers.

*Melanerpes formicivorus* (acorn woodpecker): Paramedullary diverticula are absent in the acorn woodpeckers.

#### 4.1.13 Podicipediformes

*Aechmorphus occidentalis* (Western grebe): Paramedullary diverticula are absent in Western grebes.

*Aechmorphus clarkii* (Clarke's grebe): Paramedullary diverticula are absent in Clarke's grebes.

#### 4.1.14 Procellariiformes

*Puffinus griseus* (sooty shearwater): In the sooty shearwater, paramedullary airways are present in the cervical and dorsal vertebrae, but absent in the more posterior regions. In the cervical vertebrae these structures first appear mid‐neck as a single, C‐shaped tube dorsal and lateral to the spinal cord. They are intermittently continuous and discontinuous (morphologies ii and iii) throughout the rest of the cervical series. In the anterior dorsal vertebrae, paramedullary airways are present as large, paired tubes that frequently merge to form a C‐shaped tube. They are discontinuous and connected to the cervical air sacs. In the mid‐dorsal vertebrae, the size of these diverticula is reduced and they are connected directly to diverticula from the lungs. They are absent in the posterior dorsals.

#### 4.1.15 Psittaciformes

*Pyrrhura molinae* (green‐cheeked conure): In the green‐cheeked conure paramedullary diverticula are present throughout the cervical and dorsal vertebrae, appear variably in the posterior sacral vertebrae, and are absent in all free caudals. In the cervicals, paramedullary diverticula are pared, discontinuous, and quite small. They are only present intermittently throughout this region. In the dorsal vertebrae, they become much more substantial. They expand to form a larger pair of canals that sometimes contact at the mid‐line to form a C‐shaped structure. Anteriorly, they remain discontinuous but do invade the vertebral canal (morphology ii). In mid‐ and posterior dorsals, these diverticula become continuous (morphology iii) but narrow substantially mid‐canal. Overall, the paramedullary diverticula of this region are much larger than in the cervical vertebrae. In one individual, there were small, paired canals in the last two sacral vertebrae.

*Eclectus roratus* (eclectus parrot): Paramedullary diverticula are very large in the eclectus parrot, and were observed throughout the cervical and dorsal vertebrae, as well as the posterior sacrals. They are absent in the caudal vertebrae. In the neck, the atlas, axis, and C3 are all encircled by a thick layer of paramedullary diverticula. This morphs into paired, discontinuous diverticula throughout the rest of the cervical series (morphology ii). In the thoracic region, these structures become substantially enlarged, forming a fat, single, tube dorsal to the spinal cord and continuous through the region (morphology iii). This is primarily connected to diverticula branching directly from the lungs, though anteriorly there are also connections to the cervical air sacs. We also noted one dorsal vertebral arch that was pneumatized by paramedullary airways via a foramen in the roof of the vertebral canal. These diverticula disappear in the synsacrum, but briefly reappear as large, paired tubes in the last two sacral vertebrae.

#### 4.1.16 Strigiformes

*Bubo virginianus* (great‐horned owl): In the great‐horned owl, paramedullary airways are present in all cervical vertebrae (except the atlas and axis) as paired, discontinuous tubes that invade the vertebral canal (morphology ii). In the thoracic region, this morphology persists with a moderate reduction in the size of the canals, sometimes merging at the midline to form a C‐shaped canal. They are absent through most sacral vertebrae, but are present as tiny, paired tubes lateral to the spinal cord at the very end of the synsacrum. Paramedullary diverticula are absent in the caudal vertebrae. Notably, the appearance of these structures was very similar between the two adults and individual pin‐feathered chick that were included in the study.

#### 4.1.17 Struthioniformes

*Struthio camelus* (ostrich): A whole‐body CT scan was only available for a downy ostrich chick, though paramedullary diverticula are already prominent and elaborate in the cervical region even at this relatively early ontogenetic stage. Excepting the atlas and axis, they are present in all other vertebrae of this region. Most commonly, they exist as paired tubes dorsal to the cord, which merge to form a C‐shaped canal posteriorly. They are continuous through consecutive vertebrae (morphology iii) and occasionally merge with supravertebral diverticula (morphology iv). Posteriorly, this morphs into discontinuous paired tubes (morphology ii). In the dorsal vertebrae, this morphology persists with intermittent connections to the supravertebral diverticula at intervertebral joints. Anteriorly they are paired, ovoid structures. In the mid‐ and posterior dorsals three canals dorsal and lateral to the spinal cord appear.

## 5 DISCUSSION

In this study, we find that variation of paramedullary diverticula is much greater than previously described, though notably O'Connor (2006) does make brief mention of morphological disparity among taxa. However, most previous publications (based on observations in individual taxa) describe these structures as diverticular invasions of the vertebral canal that sit as one or two tubes dorsal to the spinal cord. Incorporating data from a phylogenetically broad collection of taxa, we conclude that this definition is not inclusive of the true range of variation. Often intertransverse diverticula give off branches that contact the spinal cord at intervertebral joints, but the extent and source of invasion of the vertebral canal is highly variable. Because the degree of canal extension is not discrete, and instead is seen as a spectrum of varying levels of intrusion, we propose that all variants be considered paramedullary diverticula. Additionally, while diverticula in contact with the spinal cord are often dorsal to it, we observed many cases where these diverticula were lateral or ventral to the cord, and even several instances where the cord was surrounded by diverticula on all sides.

Paramedullary diverticula in the cervical region are connected to the cervical air sacs, as are paramedullary diverticula in the anterior dorsal vertebrae. In mid‐ and posterior dorsal vertebrae, paramedullary diverticula arise directly from diverticula branching from the lungs. Sacral and caudal paramedullary diverticula were generally quite rare and small. It was difficult to determine the origin of the structures in all cases, though we did observe in several taxa that they were connected to perirenal diverticula. Thus, we have at least partial answers to the two of the questions posed at the start of this study. Firstly, paramedullary airways share connections with both intertransverse diverticula and other extravertebral diverticula (e.g., supravertebral, perirenal). Secondly, data here indicate that cervical air sacs and diverticula often share connections with paramedullary diverticula in anterior dorsal vertebrae (when present), but not in any more posterior regions of the vertebral column.

The adaptive function of these structures—if one exists—is difficult to ascertain. Their loss in some pursuit divers (loons and grebes) appears adaptive; however, the presence of such diverticula in other pursuit divers (common murres and cormorants) seems to contradict this.

We also find that the size, complexity, and presence or absence of paramedullary diverticula varies strongly with vertebral region (Figure 3). Across the clades observed, there is a trend toward decreasing the size and presence of paremedullary diverticula moving anteroposteriorly through the vertebral column. All birds possessing paramedullary diverticula had them in the cervical region, and in all cases but one (green‐cheeked conures) they are most substantial in this part of the vertebral column. The diverticula persist into the thoracic region in only about half of observed taxa, and are present in the synsacrum and free caudals as minute pockets of air in only a handful of genera included in this study. However, we also note that it was exceedingly rare to observe paramedullary diverticula in the anterior and posterior extremes of the vertebral column. They were found in the atlas and axis of only two genera (the violet turaco and eclectus parrot), and in the pygostyle or caudal vertebrae in the violet turaco, pelican, and (inconsistently) the red‐tailed hawk.

We hypothesize that this trend may be related to varying demands of mobility in different vertebral regions. There is a clear correlation between degree of movement and the size (and presence) of paramedullary diverticula; in birds, the greatest range of motion is within the neck, where diverticula associated with the spinal cord are largest and present in the most taxa, while the vertebrae become increasingly fused and modular moving to the posterior end of the body, where paramedullary diverticula are most commonly absent. Here, paramedullary diverticula may be functioning to cushion the spinal cord as it is jostled around within the vertebral canal, functioning in a similar way to extra‐dural adipose tissue in the vertebral canal in mammals (Beaujeux et al., 1997; Megan Sions et al., 2018; Reina et al., 2009).

However, we also note that the large swelling of the spinal cord within the sacrum, known as the glycogen body (Watterson, 1949), may simply not leave any space for diverticula to invade the canal in this region. Thus, observed vertebral variation in paramedullary diverticula may be both adaptive and a corollary of spinal cord structure and development. It is also entirely possible that paramedullary diverticula simply fill in any intravertebral space that happens to be available to them, consistent with Witmer's (1997) hypothesis that diverticula are opportunistic pneumatizing machines, though this is a question for future study.

Additionally, a function of cushioning the spinal cord during neck movement makes the absence of such diverticula in woodpeckers (representative piciiforms in this study) all the more puzzling. Strong phylogenetic signal for the presence or absence of these structures as reported here indicates that phylogenetic affinity is likely a strong determining factor in whether or not a particular taxon has paramedullary airways. The presence or absence of these structures may not be adaptive at all, though taxa that *do* possess them may be exapting these diverticula to cushion the spinal cord in regions of strong vertebral movement.

### 5.1 Skeletal traces of paramedullary diverticula

**FIGURE 7:**
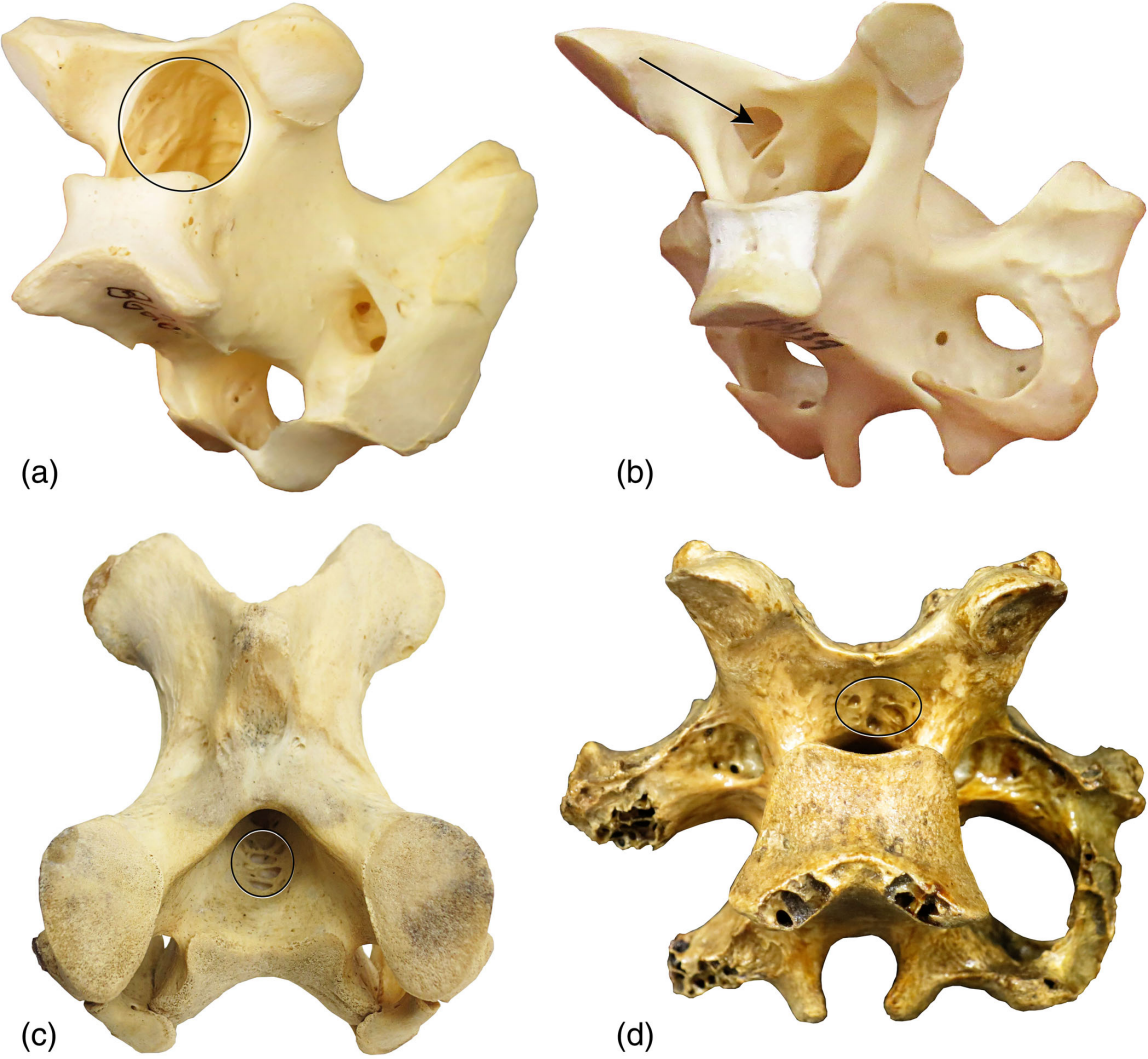
Osteological evidence of paramedullary diverticula. (a) Pocked texturing inside the vertebral canal of a pelican (LACM 86262). (b) Pneumatic foramen on the roof of the vertebral canal of an albatross (*Phoebastria nigripes*, LACM 115139). (c) Pneumatic foramina in the floor of the vertebral canal of an ostrich (Struthio camelus, LACM 116205). (d) Deep pneumatic fossae in the roof of the vertebral canal of an eastern moa (*Emeus* sp., LACM unnumbered)

In this survey we identified two osteological correlates of paramedullary diverticula (Figure 7): (a) pocked texturing of the bone in the vertebral canal where pneumatic tissue was in contact with diverticula; and (b) pneumatic foramina inside the vertebral canal, which allowed connections between the paramedullary diverticula and the interosseous diverticula that fill the vertebral central and neural arches in most birds. Across all birds examined, texturing of the bone of the vertebral canal was rare, and always occurred on the roof of the canal. Foramina were more common by comparison, and are most often formed in the dorsolateral aspect of the neural canal (but they can occur on the lateral and even the ventral aspect of the canal). Foramina in the ventral floor of the neural canal appear to be especially common in pelicans, based on observations in both dry skeletons and CT scans of multiple individuals. Importantly, both foramina and texturing are provide clues regarding the position of the paramedullary diverticula relative to the spinal cord. For instance, our preliminary data suggest that a foramen in the floor of the canal results from pneumatization via diverticula entirely jacketing the spinal cord, or would at least imply ventrally located diverticula.

Paramedullary diverticula do not always produce texturing or foramina in the walls of the neural canal. In fact, these seem to be the exception rather than the rule in most birds, with the possible exceptions of pelicans, albatrosses, ostriches, and rheas. It is probably not a coincidence that those are all large‐bodied birds with postcranial skeletons that are hyperpneumatic (sensu O'Connor, 2004). To date we have only observed one instance of pneumatic foramina inside the neural canal of sacral vertebrae (the violet turaco), and no instances of foramina inside the canals of caudal vertebrae. All other occurrences of pneumatic foramina inside the canal were seen in presacral vertebrae. It is tempting to infer a biological basis, given that the cervical and dorsal vertebrae are the regions of the postcranial skeleton that are most commonly pneumatized at all in both extant birds (O'Connor, 2006), extinct non‐avian theropods (Benson et al., 2012), pterosaurs (Claessens et al., 2009), and sauropods (Wedel, 2009). The apparent restriction of neural canal foramina to the presacral vertebrae could be an artifact of sampling; however, given that such foramina appear to be rare outside of the aforementioned hyperpneumatic taxa, and that paramedullary diverticula do not exist in the sacral and free caudal vertebrae of most birds. Even a random distribution of neural canal foramina would lead to most examples occurring in cervical and dorsal vertebrae, since that is where the paramedullary diverticula themselves are most commonly located. Our own sampling of dry, osteological specimens is very limited, more of an exploration of the morphologies realized in extant birds than a systematic survey that could elucidate statistical regularities. This is an area in which anyone with access to an osteological collection of extant birds could make important contributions with relatively little effort.

In sum, although paramedullary diverticula can form texturing or pneumatic foramina in the walls of the neural canal, more commonly they do not leave any diagnostic skeletal trace at the level of gross visual examination—in other words, they are cryptic diverticula (sensu Wedel & Taylor, 2013). Even in the absence of gross osteological correlates, paramedullary diverticula might still leave distinct histological traces, such as the “pneumosteum” identified by Lambertz et al. (2018); this is another area that is ripe for further investigation.

## 6 CONCLUSIONS AND DIRECTIONS FOR FUTURE RESEARCH

In this study we have cast a broader net, phylogenetically speaking, than any previous work on paramedullary airways, but there is still much to be done. We were only able to assess a handful of individuals at most for any given species, and for several species only a single individual. Our sampling of species within genera and genera within larger clades is likewise limited. Finally, with so few individuals in the study, our ontogenetic sampling is poor. Further studies addressing the ontogenetic development of paramedullary diverticula, their intraspecific variation, and their clade‐level diversity would all be welcome advances.

Much interesting work remains to be done on the basic anatomy of paramedullary diverticula. On a fine scale, it would be useful to know if the pneumatic epithelium that lines the diverticula is firmly attached, either to periosteum that lines the neural canal or to the dura mater, or if the paramedullary diverticula are potentially mobile within the neural canal. As discussed above, further documenting the connections of the paramedullary diverticula to specific air sacs or specific portions of the lungs might elucidate both the development and evolution of this system. Furthermore, the physiological implications of paramedullary diverticular, if any, are completely unknown. Is there a possibility for active circulation of air through an anastomosing system of paramedullary diverticula? Could these diverticula function in either insulating or cooling the central nervous system? These questions await investigation by clever physiologists.

Turning to osteological correlates, surveying the neural canals of skeletonized birds in museum collections for pneumatic sculpturing or foramina could potentially yield much useful information for a relatively small investment of effort. A better foundation of skeletal evidence will be needed to determine if the extent of paramedullary diverticula is correlated with degree of skeletal pneumatization. It may be possible to map the distribution of paramedullary diverticula using bone histology—even determining if this is possible in a chicken or a turkey would be valuable contribution.

The quest to document paramedullary diverticula need not be limited to extant archosaurs. The presence of pneumatic fossae or foramina in the neural canal of an Eastern moa, *Emeus* sp. (Figure 7d), demonstrates the potential for finding evidence of paramedullary diverticula in extinct birds. If present, skeletal traces of paramedullary diverticula should be particularly easy to identify in the vertebrae of large flightless (extinct ratites, phorusrhacids, *Diatryma*) and flighted (pelegornithids, teratorns) birds.

In addition, an avian‐like respiratory system is known to have been present in several lineages of extinct archosaurs as well, including saurischian dinosaurs (O'Connor & Claessens, 2005; Wedel, 2003a, 2009) and pterosaurs (Claessens et al., 2009). Extensive pneumatization of the vertebral column in these clades shows that vertebral diverticula were present (Benson et al., 2012; Schwarz et al., 2007; Wedel, 2003b), and the clustering of pneumatic features around the neural canal hints that paramedullary diverticula may have been present even when there is no direct evidence for them (Schwarz & Fritsch, 2006; Taylor & Wedel, 2021). To date, the only described evidence for paramedullary diverticula specifically in non‐avian archosaurs are pneumatic foramina connecting the neural canals to pneumatic chambers in cervical vertebrae of the brachiosaurid dinosaur *Giraffatitan* (Schwarz & Fritsch, 2006), and in a dorsal vertebra of an unnamed saltasaurid sauropod (Aureliano et al., 2021). Such foramina are not easy to detect, because the neural canals of fossil vertebrates are rarely completely prepared out (i.e., with the rock matrix removed from the canal). The canals can be difficult to examine even when they are completely prepared, and the number of vertebrae that have been CT scanned is small. Furthermore, as discussed above, even in most birds in which they occur, paramedullary diverticula do not leave foramina or other diagnostic skeletal traces. If the same was true in extinct archosaurs, small sample sizes may hinder the quest for more examples of paramedullary diverticula in the fossil record but is a line of inquiry that should be pursued nonetheless.

As this study shows, paramedullary diverticula in birds exhibit a much broader range of morphologies than previously reported or suspected. Sampling issues notwithstanding, we predict that if more effort is directed to finding evidence of paramedullary diverticula in fossil taxa, more will be found, increasing both the number and morphological variety of known examples. We hope that this study serves as a foundation and an enticement for further studies of this most unusual anatomical system, in both extinct and extant archosaurs.

## CONFLICT OF INTEREST

The authors have no conflicts of interest.

## AUTHOR CONTRIBUTIONS

**Jessie Atterholt:** Conceptualization (supporting); data curation (lead); formal analysis (equal); funding acquisition (lead); investigation (equal); methodology (equal); project administration (lead); resources (equal); software (equal); supervision (equal); writing – original draft (lead); writing – review and editing (equal). **Mathew Wedel:** Conceptualization (lead); data curation (supporting); formal analysis (equal); funding acquisition (equal); investigation (equal); methodology (equal); project administration (supporting); resources (equal); software (equal); supervision (equal); writing – original draft (supporting); writing – review and editing (equal).

## Data Availability

All microCT scans used as data in this study are openly available on MorphoSource, and can be found under the project “Atterholt Dissertation.” Scans collected on a conventional scanner are shared data with another research group. These colleagues have on‐going studies using the data, and have requested that the scans not be made publicly available at this time.
